# Establishment and validation of a nomogram to predict cancer-specific survival in pediatric neuroblastoma patients

**DOI:** 10.3389/fped.2023.1105922

**Published:** 2023-03-03

**Authors:** Weiming Chen, Ping Lin, Jianxi Bai, Yifan Fang, Bing Zhang

**Affiliations:** ^1^ Department of Pediatric Surgery, Fujian Children’s Hospital (Fujian Branch of Shanghai Children’s Medical Center), College of Clinical Medicine for Obstetrics & Gynecology and Pediatrics, Fujian Medical University, Fuzhou, China; ^2^ Department of Hematology and Oncology, Fujian Children’s Hospital (Fujian Branch of Shanghai Children’s Medical Center), College of Clinical Medicine for Obstetrics & Gynecology and Pediatrics, Fujian Medical University, Fuzhou, China

**Keywords:** neuroblastoma, cancer-specific survival, nomogram, SEER, prognosis

## Abstract

**Background:**

The term “neuroblastoma (NB)” refers to a type of solid pediatric tumor that develops from undivided neuronal cells. According to the American Cancer Society report, between 700 and 800 children under the age of 14 are diagnosed with NB every year in the United States (U.S.). About 6% of all cases of pediatric cancer in the U.S. are caused by NB. NB is the most frequent malignancy in children younger than 1 year; however, it is rarely found in those over the age of 10 and above.

**Objective:**

To accurately predict cancer-specific survival (CSS) in children with NB, this research developed and validated an all-encompassing prediction model.

**Methods:**

The present retrospective study used the Surveillance, Epidemiology, and End Results (SEER) database to collect information on 1,448 individuals diagnosed with NB between 1998 and 2019. The pool of potentially eligible patients was randomly split into two groups, a training cohort (*N* = 1,013) and a validation cohort (*N* = 435). Using multivariate Cox stepwise regression, we were able to identify the components that independently predicted outcomes. The accuracy of this nomogram was measured employing the consistency index (C-index), area under the time-dependent receiver operating characteristic curve (AUC), calibration curve, and decision-curve analysis (DCA).

**Results:**

In this study, we found that age, primary location, tumor size, summary stage, chemotherapy, and surgery were all significant predictors of CSS outcomes and integrated them into our model accordingly. The C-index for the validation cohort was 0.812 (95% CI: 0.773–0.851), while for the training cohort it was 0.795 (95% CI: 0.767–0.823). The C-indexes and AUC values show that the nomogram is able to discriminate well enough. The calibration curves suggest that the nomogram is quite accurate. Also, the DCA curves demonstrated the prediction model's value.

**Conclusion:**

A novel nomogram was developed and validated in this work to assess personalized CSS in NB patients, and it has been indicated that this model could be a useful tool for calculating NB patients’ survival on an individual basis and enhancing therapeutic decision-making.

## Introduction

Neuroblastoma (NB) is considered to be the most prevalent extracranial malignant solid tumor in children, originating from growing neural crest cells and affecting anywhere in the sympathetic nerves (1, 2). Neuroblastomas exhibit varied clinical behavior, ranging from spontaneous regression or differentiation into a benign ganglioneuroma to unrelenting progression despite rigorous, multimodal treatment (3). Children with neuroblastoma account for 8%–10% of all childhood cancers and nearly 15% of all pediatric cancer-related mortality (4, 5).

The current studies have systematically demonstrated various risk factors closely related to the survival of NB, such as age, surgical treatment, tumor size, radiotherapy, and chemotherapy (6–10). However, neither these independent prognostic factors for patients with NB nor the widely used International Neuroblastoma Staging System (INSS) can provide a perfect evaluation system for personalized and accurate assessment of patient survival probability. Nomograph enables a personalized assessment of the likelihood of occurrences, which is successful in a variety of malignancies, by incorporating multiple critical parameters (11). To date, only a few studies have foucus on a clinically convenient model for predicting overall survival in neuroblastoma patients (12, 13).

Using clinical data of NB patients from the SEER dataset between 1998 and 2019, an accurate model was constructed to predict the CSS rates of NB patients at 1, 3, and 5 years following diagnosis. The obtained results led to the achievement of a personalized and accurate estimation of the likelihood of survival and helped guide the decision-making process for clinical protocols. In addition, it has been suggested that this nomogram will be a useful tool for calculating personalized survival predictions for NB patients and optimizing clinical decision-making.

## Materials and methods

### Patient selection

**Figure 1:**
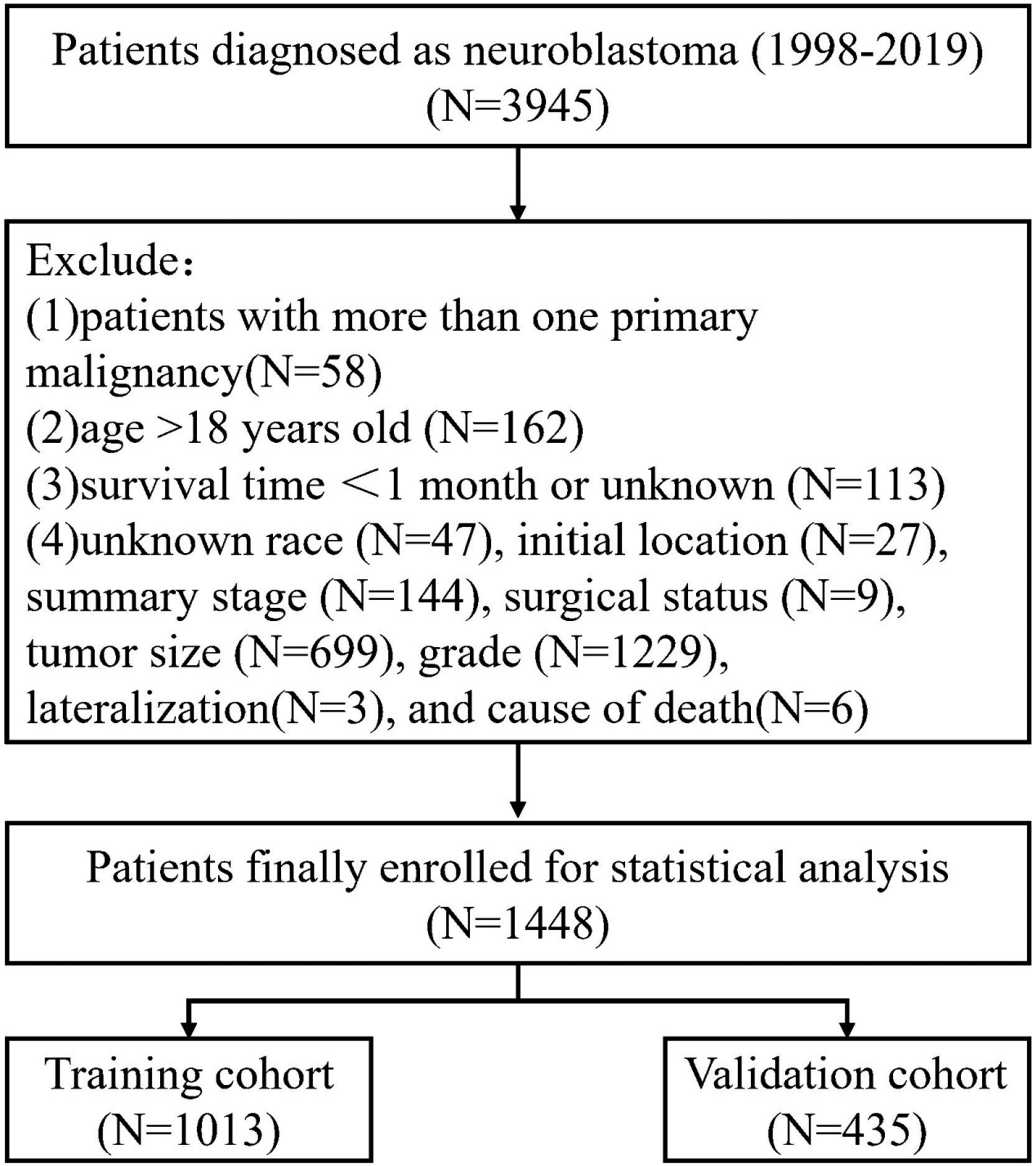
Flowchart of sample selection.

The SEER database at the National Cancer Institute was accessed through SEER*Stat version 8.4.0 to compile the data for this analysis. The SEER database includes information from eighteen population-based cancer registries, representing around 28% of the U.S. population. Youth-specific NB data were obtained from the SEER Registry (14). The following conditions were set for participation: (1) The specific histological subtype of NB (AYA site recode 2020) Neuroblastoma/ganglioneuroblastoma, Third Edition; (2) Patients Diagnosed Between 1998 and 2019; (3) Detailed Information on Follow-Up. As for the criteria for exclusion, they were as follows: Patients with less than a month to live; patients older than 18 years old; patients whose race, initial location, summary stage, tumor size, surgical status, grade, lateralization, and cause of death are unknown; and patients with more than one primary malignancy. The flowchart of sample selection is presented in Figure 1.

### Variable collection

**Figure 2:**
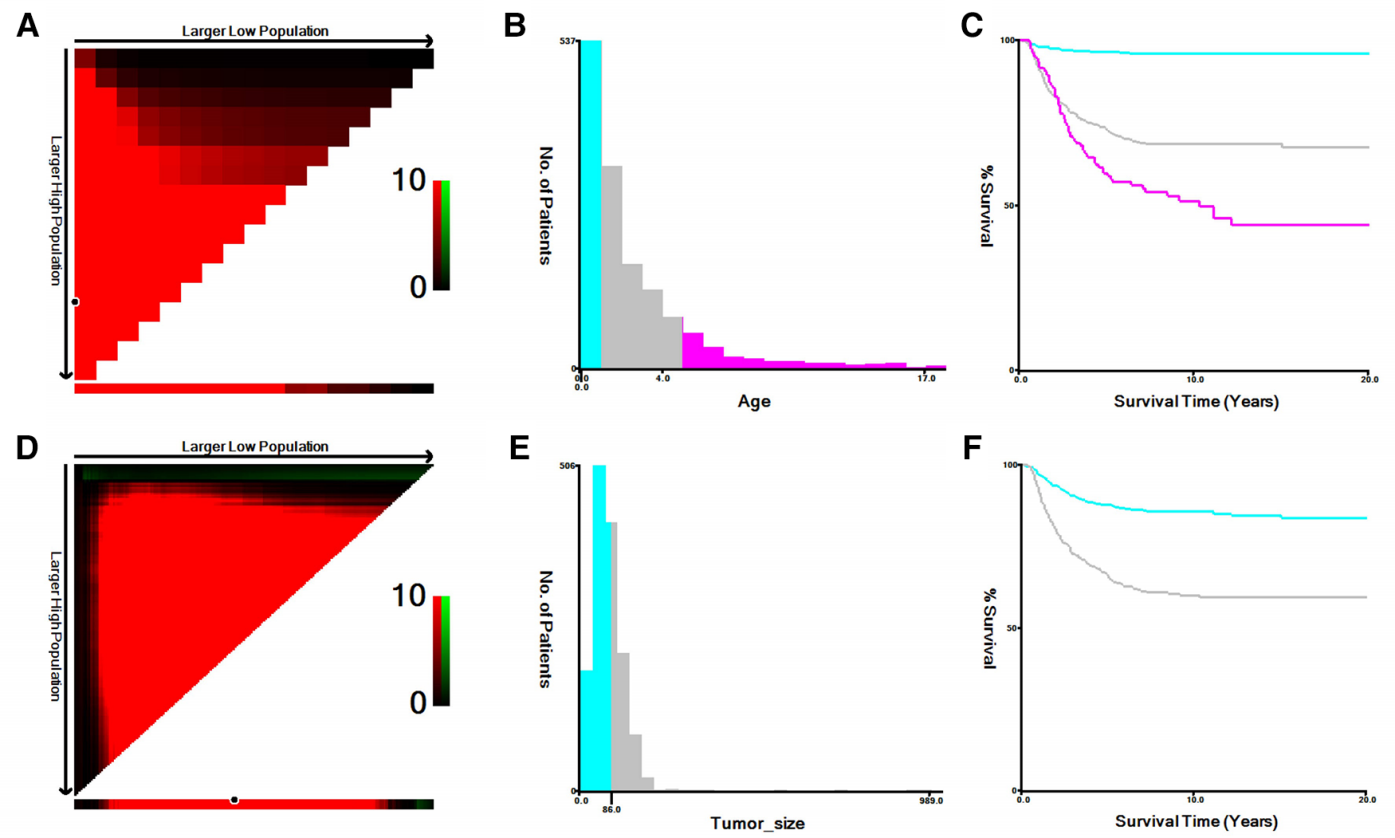
The optimal cutoff values of age and tumor size identified by X-tile. (**A,B**) The optimal cutoff value of age. (**C**) The Kaplan–Meier curves for the subgroups of age (<1, 1–5, ≥5) for CSS. (**D,E**) The cutoff value of tumor size. (**F**) The Kaplan–Meier curves for the subgroups of tumor size (<8.7 cm, ≥8.7 cm) for CSS.

Year of diagnosis, race, sex, age at diagnosis, primary site, histologic type, tumor-grade (well, moderately, poorly, and undifferentiated/anaplastic, considered grades I–IV, respectively), laterality, tumor size, surgery status, summary stage (localized, regional, and distant), radiotherapy status, cause of death, chemotherapy status, and survival time were all collected from all eligible cases. Figure 2 displays the results of using the X-tile program developed at Yale University (New Haven, CT, United States) to determine an acceptable cut-off point for classifying continuous variables. Further, tumor size was separated into two groups, <8.7 and ≥8.7 cm, while age was split into three groups: <1, 1–5, and ≥5 years.

Time from cancer diagnosis to death (CSS) was the main outcome of this research. Obtaining formal informed permission from research participants was waived since the patient data in the SEER database had been de-identified and patients had signed informed consent forms before surgery and treatment.

### Statistical evaluations

In this study, we used the chi-square test to evaluate differences in categorical variables between the data used for training and the data used for validation. Kaplan-Meier estimates and log-rank tests were used to identify the univariate prognostic factors of CSS. Multivariate Cox analysis was used to predict CSS outcomes, and components with *p*-values <0.05 were judged to be autonomous. Using these independent prognostic markers, a nomogram was created for predicting 1-, 3-, and 5-year CSS in children with neuroblastoma. C-index and ROC curve analysis were used to evaluate the established nomogram's discriminatory power. From 0.5 (no discrimination) to 1 (perfect discrimination), this was the range of values for the C-index statistic (15). Through the use of calibration curves, we were able to evaluate the consistency between the estimated and observed probabilities. Afterward, decision curve analysis was used to assess the nomogram's overall clinical utility (DCA). Furthermore, SPSS Ver-26.0 (IBM Corporation, United States) and R version-4.2.0 (www.r-project.org) were applied for the data assessments. Moreover, a *p*-value < 0.05 was deemed statistically significant.

## Results

### Baseline characteristics

**Figure 3:**
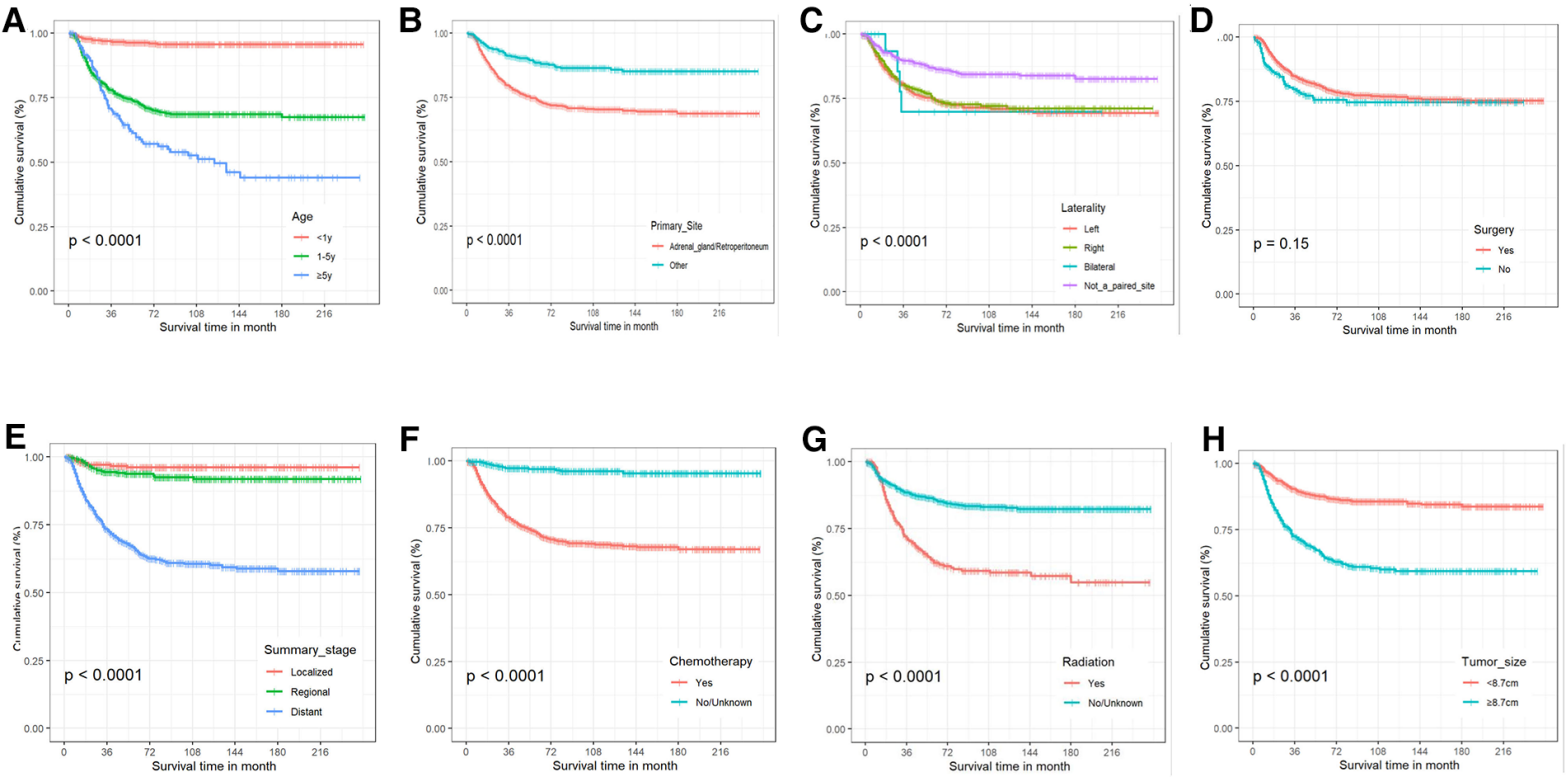
The Kaplan-Meier curves for cancer-specific survival. (**A**) Age. (**B**) Primary tumor site. (**C**) Tumor laterality. (**D**) Surgery. (**E**) Summary stage. (**F**) Chemotherapy. (**G**) Radiotherapy. (**H**) Tumor size.

Patients with NB that satisfied the above criteria numbered 1,448, and they were split into a training group of 1,013, and a validation cohort of 435, in a 7:3 ratio. Table 1 presents the basic sociodemographic and clinicopathological characteristics of the patients in the whole cohort, the training cohort, and the validation cohort. Differences in baseline data between the two groups were not statistically significant and were verifiable by a third party. The overall 1-, 3-, and 5-year survival rates for the whole cohort were 94.3%, 84.0%, and 79.6%, respectively; the median follow-up duration for the 1,448 children with neuroblastoma was 54 months. The 5-year CSS for this group of NB patients was 65.5%, and 52.8% of them had distant metastases. Figure 3 demonstrates the survival curves for age, tumor grade, primary tumor site, tumor laterality, surgery, summary stage, radiotherapy, chemotherapy, and tumor size. The results of KM analysis showed that all eight variables were linked with the prognosis of neuroblastoma (*p *< 0.001) except for surgery (*p *= 0.15).

**Table 1:** Demographic characteristics and clinicopathological features of 1,448 NB patients.

Variables	Whole cohort (*N* = 1,448), *n* (%)	Training cohort (*N* = 1,013), *n* (%)	Validation cohort (*N* = 435), *n* (%)	*p*
**Age**				0.812
<1	537 (37.1)	380 (37.5)	157 (36.1)	
1–5	715 (49.4)	499 (49.3)	216 (49.7)	
≥5	196 (13.5)	134 (13.2)	62 (14.3)	
**Gender**				0.389
Male	784 (54.1)	556 (54.9)	228 (52.4)	
Female	664 (45.9)	457 (45.1)	207 (47.6)	
**Race**				0.072
White	1,121 (77.4)	794 (78.4)	327 (75.2)	
Black	190 (13.1)	135 (13.3)	55 (12.6)	
Other	137 (9.5)	84 (8.3)	53 (12.2)	
**Primary site**				0.478
Adrenal gland/Retroperitoneum	901 (62.2)	624 (61.6)	277 (63.7)	
Other	547 (37.8)	389 (38.4)	158 (36.3)	
**Histology**				0.654
Neuroblastoma	1,347 (93.0)	940 (92.8)	407 (93.6)	
Ganglionneuroblastoma	101 (7.0)	73 (7.2)	28 (6.4)	
**Grade**				0.114
I–II	83 (5.7)	62 (6.1)	21 (4.8)	
III	1,142 (78.9)	784 (77.4)	358 (82.3)	
IV	223 (15.4)	167 (16.5)	56 (12.9)	
**Laterality**				0.596
Left	491 (33.9)	342 (33.8)	149 (34.3)	
Right	388 (26.8)	279 (27.5)	109 (25.1)	
Bilateral	20 (1.4)	12 (1.2)	8 (1.8)	
Not a paired site	549 (37.9)	380 (37.5)	169 (38.9)	
**Summary stage**				0.258
Localized	276 (19.1)	203 (20.0)	73 (16.8)	
Regional	408 (28.2)	288 (28.4)	120 (27.6)	
Distant	764 (52.8)	522 (51.5)	242 (55.6)	
**Surgery**				0.812
Yes	1,224 (84.5)	858 (84.7)	366 (84.1)	
No	224 (15.5)	155 (15.3)	69 (15.9)	
**Radiotherapy**				0.949
Yes	412 (28.5)	289 (28.5)	123 (28.3)	
No/Unknown	1,036 (71.5)	724 (71.5)	312 (71.7)	
**Chemotherapy**				0.403
Yes	1,053 (72.7)	730 (72.1)	323 (74.3)	
No/Unknown	395 (27.3)	283 (27.9)	112 (25.7)	
**Tumor size**				0.312
<8.7 cm	918 (63.4)	651 (64.3)	267 (61.4)	
≥8.7 cm	530 (36.6)	362 (35.7)	168 (38.6)	
**Status**				0.081
Alive	1,170 (80.8)	831 (82.0)	339 (77.9)	
Dead	278 (19.2)	182 (18.0)	96 (22.1)	
**CSS [mean (SD)]**	78.82 (65.60)	80.67 (66.03)	74.51 (64.45)	0.101

### Prognostic factors of CSS

The results of the univariate analysis showed that the prognosis of children with neuroblastoma was influenced by factors such as age, primary location, grade, laterality, summary stage, radiation, chemotherapy, and tumor size (all *p* values <0.05). Table 2 displays the results of a multivariate Cox regression analysis, which showed that age, primary site, summary stage, surgery, treatment, and tumor volume were all independent variables impacting the prognosis of NB patients (all *p* values <0.05).

**Table 2:** Univariate and multivariate Cox regression analysis for cancer-specific survival in patients with neuroblastoma.

Variables	Univariate analysis			Multivariate analysis		
	HR	95% CI	*p*	HR	95% CI	*p*
**Age**						
<1 year		Reference			Reference	
1–5 years	7.182	4.199–12.280	<0.001	4.334	2.418–7.768	<0.001
≥5 years	11.141	6.215–19.970	<0.001	5.849	3.104–11.019	<0.001
**Gender**						
Male		Reference			Reference	
Female	1.134	0.8473–1.518	0.398	1.256	0.934–1.690	0.132
**Race**						
White		Reference			Reference	
Black	1.191	0.7932–1.789	0.399	0.935	0.618–1.413	0.748
Other	1.432	0.8648–2.372	0.163	1.181	0.708–1.971	0.524
**Primary site**						
Adrenal gland/Retroperitoneum		Reference			Reference	
Other	0.335	0.231–0.487	<0.001	0.573	0.368–0.892	0.014
**Histology**						
Neuroblastoma		Reference			Reference	
Ganglionneuroblastoma	1.054	0.611–1.818	0.850	1.465	0.827–2.593	0.190
**Grade**						
I–II		Reference			Reference	
III	1.928	0.787–4.726	0.151	0.749	0.290–1.937	0.551
IV	5.519	2.223–13.700	<0.001	1.474	0.557–3.900	0.435
**Laterality**						
Left		Reference			Reference	
Right	1.017	0.726–1.423	0.923	1.001	0.711–1.409	0.997
Bilateral	0.791	0.194–3.223	0.744	0.608	0.148–2.501	0.490
Not a paired site	0.472	0.324–0.686	<0.001	1.075	0.690–1.677	0.749
**Summary stage**						
Localized		Reference			Reference	
Regional	1.905	0.801–4.532	0.145	1.021	0.420–2.482	0.963
Distant	10.735	5.033–22.897	<0.001	3.574	1.572–8.126	0.002
**Surgery**						
Yes		Reference			Reference	
No	1.401	0.961–2.043	0.080	1.598	1.061–2.404	0.025
**Radiotherapy**						
Yes		Reference			Reference	
No/Unknown	0.384	0.287–0.514	<0.001	1.148	0.835–1.577	0.395
**Chemotherapy**						
Yes		Reference			Reference	
No/Unknown	0.086	0.040–0.182	<0.001	0.327	0.138–0.772	0.011
**Tumor size**						
<8.7 cm		Reference			Reference	
≥8.7 cm	3.437	2.551–4.631	<0.001	1.425	1.035–1.961	0.030

### Nomogram construction

**Figure 4:**
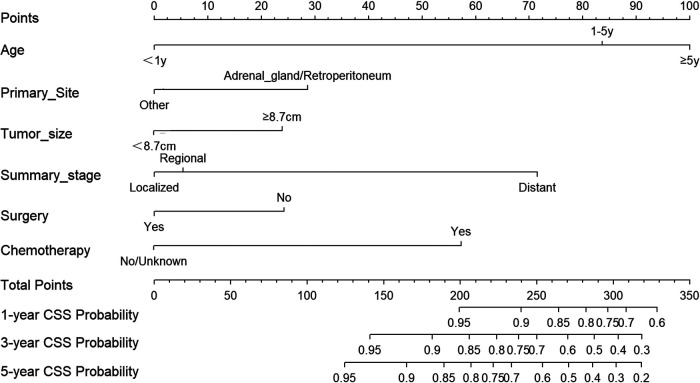
Nomogram for predicting 1-, 3-, and 5-year cancer-specific survival in NB patients.

Based on the aforementioned independent factors on the prognosis of NB patients, a prognostic prediction model was developed and presented as a nomogram to visually represent 1-, 3-, and 5-year CSS of NB, as shown in Figure 4. Age had the highest predictive effect, followed by summary stage, chemotherapy, primary site, surgery, and tumor size. By summing the scores corresponding to each particular variable, the likelihood of CSS can be simply estimated. For example, if the child was diagnosed with neuroblastoma at 2 years of age (84 points), the child would have received chemotherapy (57 points) without any surgical intervention (24 points). The primary site of the cancer was in the adrenal region (29 points), the maximum diameter of the tumor was 10 cm (24 points), and the tumor had distant metastasis (72 points). The patient would have a total score of 290 points. Thus, the probability of CSS at 1-, 3- and 5-year was 77%, 49%, and 38%, respectively.

### Assessment and validation of the nomogram

**Figure 5:**
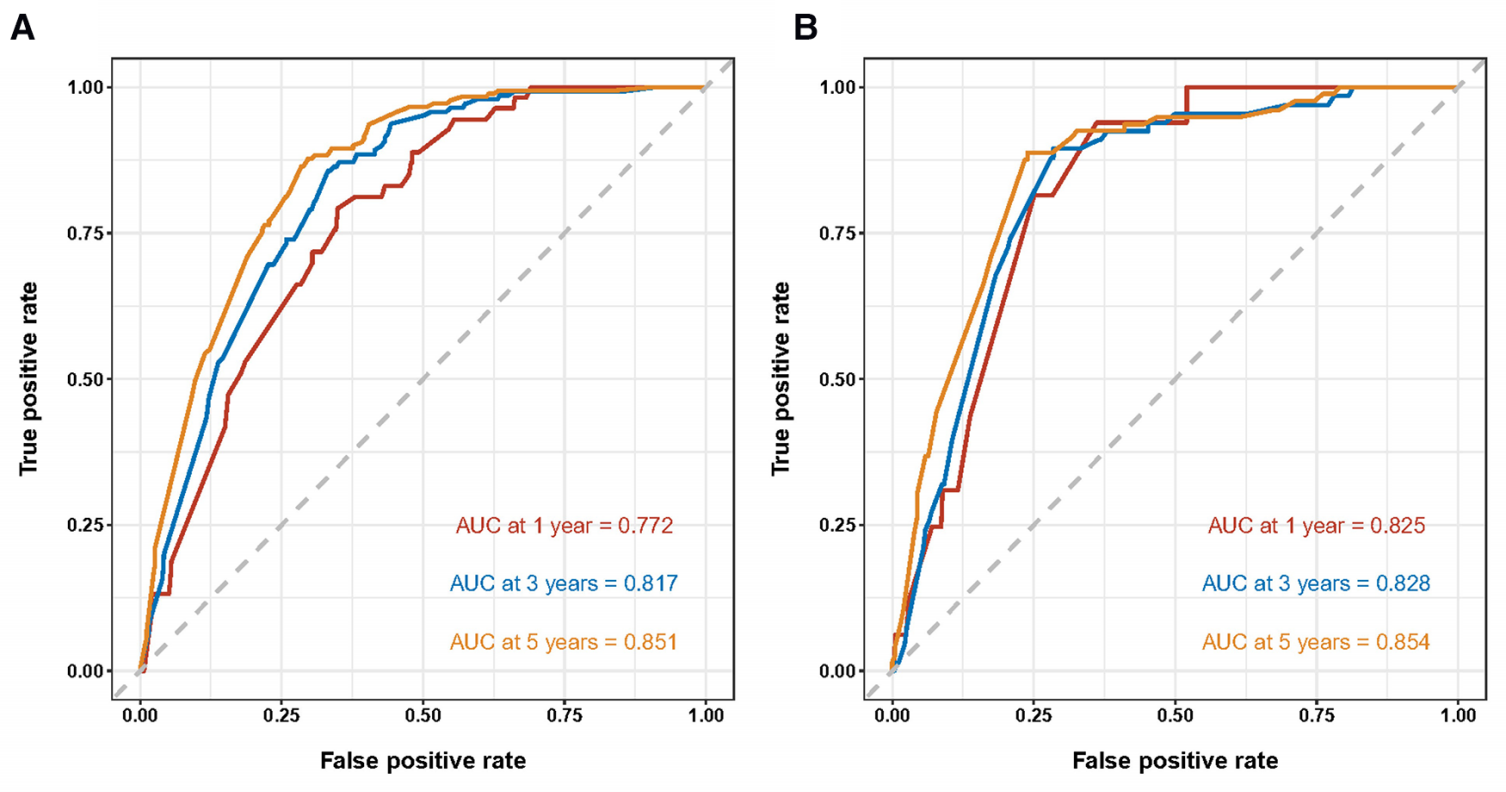
ROC curves of the nomogram for predicting 1-, 3-, and 5-year cancer-specific survival in the training cohort (**A**) and the validation cohort (**B**).

**Figure 6:**
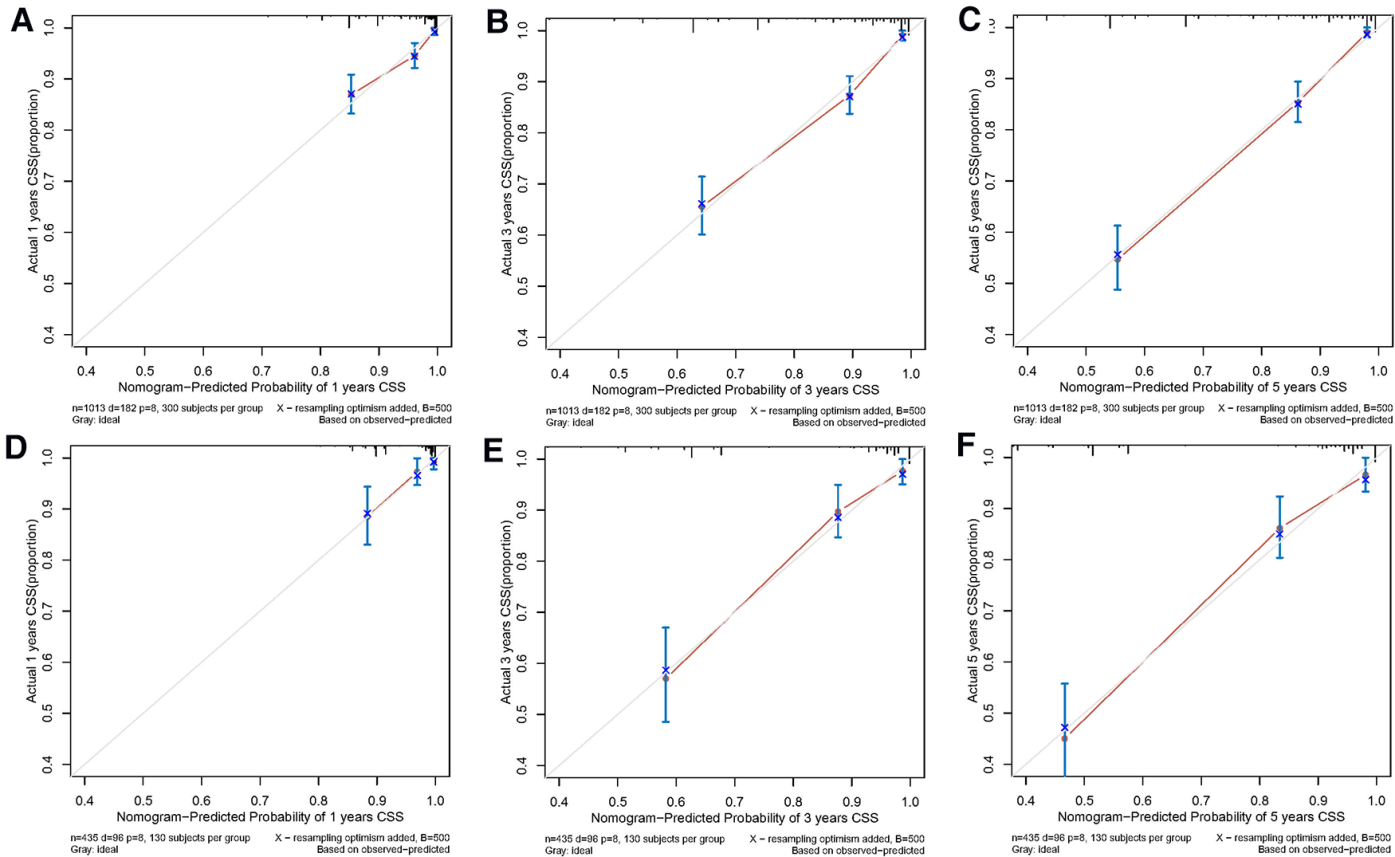
The calibration curves of the prognostic nomogram for predicting 1-year (**A**), 3-year (**B**), and 5-year (**C**) CCS in the training cohort. The alibration curves of the prognostic nomogram for predicting 1-year (**D**), 3-year (**E**), and 5-year (**F**) CSS in the validation cohort.

**Figure 7:**
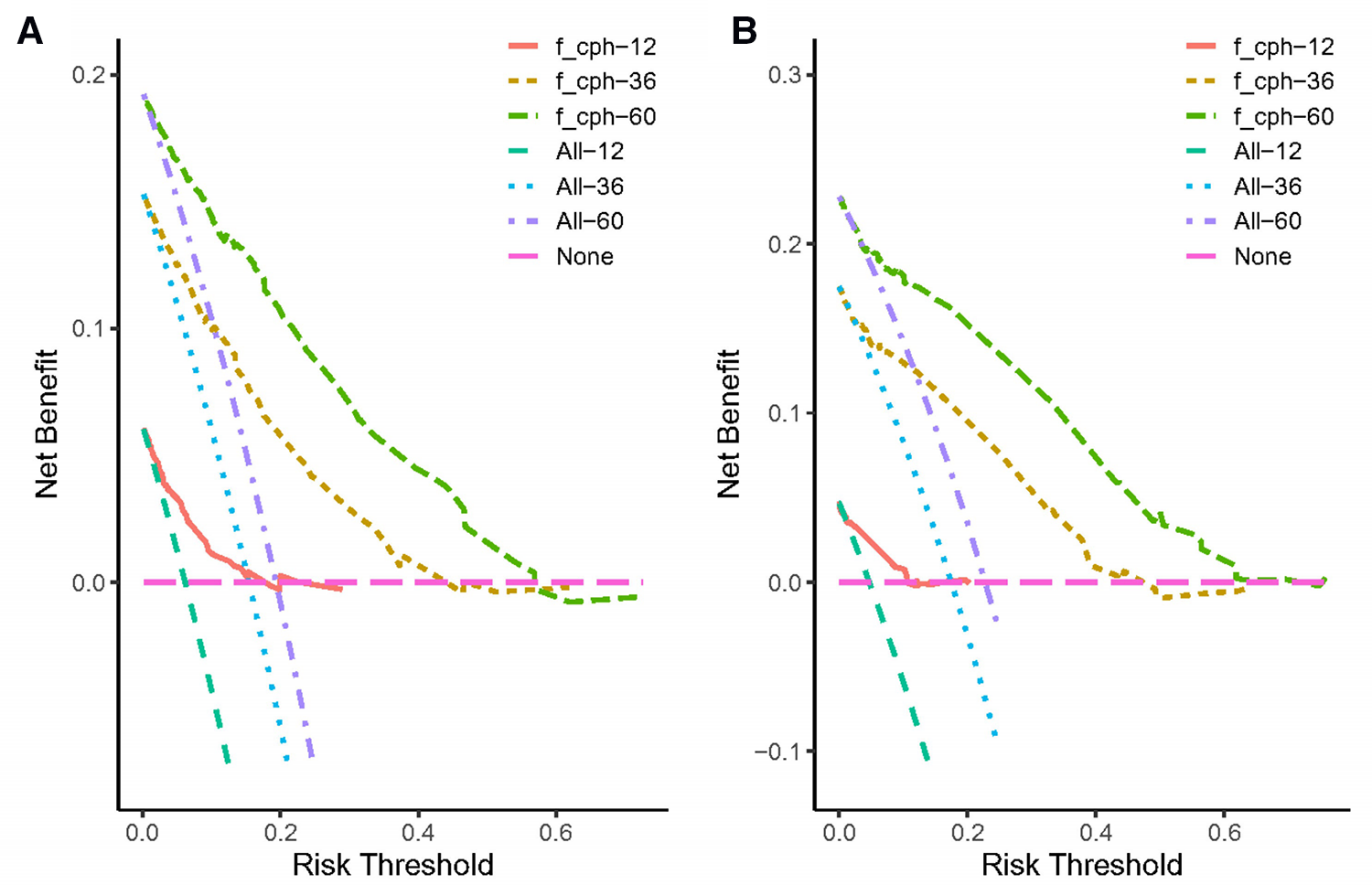
The decision curve analysis of the nomogram for predicting 1-, 3-, 5-year cancer-specific survival in the training cohort (**A**) and the validation cohort (**B**).

Both the training cohort's and the validation cohort's C-indices were 0.795 and 0.812, respectively. Figure 5 depicts the nomogram's ROC curves for predicting 1-, 3-, and 5-year CSS. In the training cohort, the AUCs for 1-, 3-, and 5-year predictions were 0.772, 0.817, and 0.851, respectively. Moreover, 0.825, 0.828, and 0.854 were the respective AUCs for the validation cohort. According to the results above, the nomogram has a strong discriminating value. The nomogram's predicted probability values and the actual probability values are represented by the calibration curve, which shows the relationship between them. The y-axis displays the actual 1-, 3-, and 5-year survival probability, whereas the x-axis displays the expected 1-, 3-, and 5-year survival probabilities of the nomogram. The calibration curves in this investigation demonstrated that there was a good correspondence between the nomogram's predicted survival probability and the actual outcomes (Figure 6). For CSS at 1-, 3-, and 5-year intervals, DCA analysis has shown that the nomogram could demonstrate substantial net benefit and clinical validity (Figure 7).

### An online tool for predicting CSS

**Figure 8:**
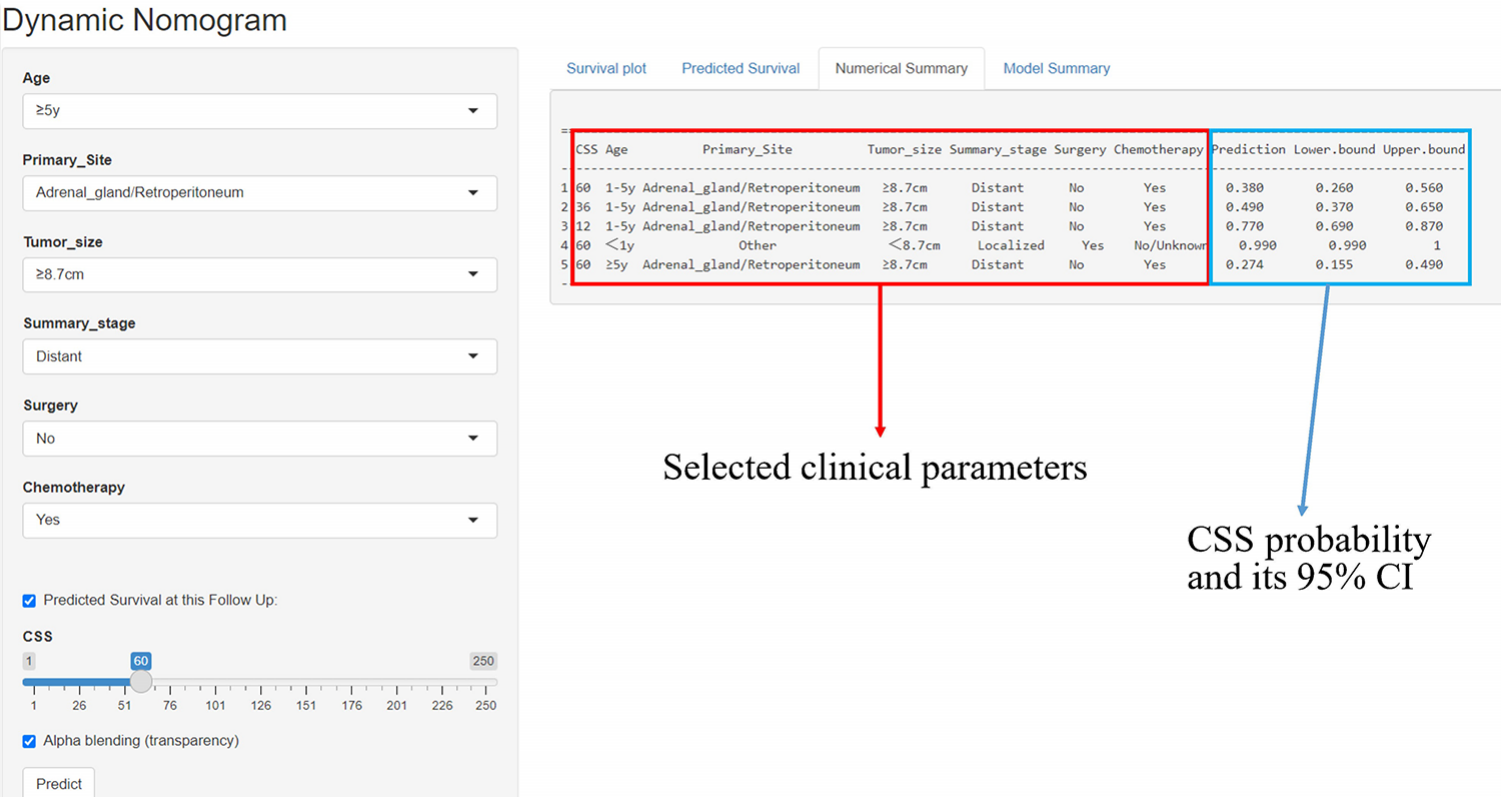
The interface of the web-based nomogram, and numerical summary of the CSS probability.

A simple online application (https://fjsrtyyxewk.shinyapps.io/DynNomapp/) was developed to visualize the nomogram. By choosing the appropriate clinical features and follow-up period, the patient's CCS curve and likelihood can be observed, as shown in Figure 8.

## Discussion

Neuroblastoma (NB) is considered to be the most prevalent and aggressive extracranial malignancy. NB is a solid tumor in children and originates from growing neural crest cells, affecting anywhere in the sympathetic nerves (1, 2). Herein, we established an accurate model for estimating the CSS rates of NB patients at 1, 3, and 5 years after diagnosis using the clinical data of NB patients from the SEER dataset between 1998 and 2019. Hence, a model was established that can predict the survival outcome of neuroblastoma patients individually. Nomograms are mathematical model plots that combine clinical and biological factors to estimate the likelihood of an incident with a specific clinical outcome. Numerous studies have demonstrated that nomograms are more accurate than conventional tumor staging systems and are frequently used in the field of oncology to predict patients’ chances of surviving and developing complications (16–18). Herein, a nomogram developed using retrospective data from the SEER database on 1,448 children with neuroblastoma was used to determine five independent predictive markers for children with neuroblastoma and to develop a nomogram that can visually and precisely predicts neuroblastoma survival. Compared with previous studies, our nomogram exhibits better discriminating and calibration capabilities, and we have developed an easy-to use online application that can be applied immediately to aid clinical decision-making (12, 13).

According to multiple reported studies, the clinical, biological, and molecular characteristics of neuroblastoma are significantly predictive of clinical outcomes (19, 20). Several studies have indicated that age is a critical determinant of the prognosis for NB patients (6, 21, 22). Sokol et al. (22) demonstrated that a diagnosis of neuroblastoma after the age of 18 months is a significant risk factor. In the current study, the X-tile tool was used to divide the age of children into three groups, i.e., <1, 1–5, and ≥5 years, with children <1 year having a higher CSS and patients ≥5 years having the worst CSS. The obtained results suggested that the proportion of children diagnosed at ≥1 year of age is about 63%, which may be related to the insidious onset of NB, the diversity and lack of specificity of clinical presentation, and the lack of screening criteria and protocols for malignancy.

In this data set, about 62% of children with neuroblastoma were found to have the disease in the retroperitoneum and adrenal glands. These children have a worse prognosis than those whose tumors are present in other locations. Neuroblastoma can develop in the retroperitoneal sympathetic ganglion and adrenal medulla (23). At the time of NB diagnosis, about 40% of patients have distant metastases. The 5-year survival rate for patients with NB in the high-risk group is still less than 50% worldwide and even less than 40% in China, despite the fact that current treatment regimens are being improved and intensified (24). These findings were found similar to our study, which showed that NB patients with distant metastases have worse CSS. In this study, around 53% of NB patients had distant metastases, and the 5-year cancer-specific survival rate of NB patients in the metastatic group was 65.5%. Additionally, NB indicates rapid proliferation and development; in this study, 37% of the tumors had a maximal diameter greater than 8.7 cm. Children with greater tumor sizes (≥8.7 cm), according to the results of multifactorial Cox regression, had a worse prognosis.

Current treatment modalities for NB involve multiple disciplines, including surgery, chemotherapy, and radiation therapy (25). Surgery is a significant prognostic factor (26). However, the effect of the degree of tumor excision on the prognosis of children with NB is currently the subject of debate (27, 28). In the present study, children were categorized according to whether or not they had undergone surgical intervention (regardless of the surgical modality), and the univariate Cox regression and KM survival analysis showed no statistically significant difference in prognosis between the surgical and non-surgical groups. In contrast, in the multifactorial cox regression model, failure to undergo surgical treatment was indeed an independent factor influencing poor prognosis in neuroblastoma. However, in children with advanced NB, considering the presence of surgical risk factors such as extensive tumor involvement and tumor encapsulation of important blood vessels, aggressive preoperative chemotherapy is required, and surgery is performed at a reasonable time after tumor shrinkage (29). In our study, the results of KM survival analysis showed that children in the radiotherapy group had a poorer prognosis, and radiotherapy may be a poor prognostic factor for NB in the univariate analysis. These results may be covariate with the fact that the majority of children with advanced NB require radiotherapy. Hence, in the univariate study, radiotherapy was incorrectly identified as a factor contributing to poor prognosis in NB. However, in the multifactorial Cox regression analysis, radiotherapy was not identified as an independent prognostic factor. In addition, chemotherapy was significantly associated with poor prognosis in the univariate analysis as well as the multivariable model. This was consistent with results by Liang et al. (12).

In this study, a nomogram was generated for the prediction of 1-, 3-, and 5-year CSS in NB patients based on a retrospective examination of the SEER database using a multifactor Cox regression model. The prediction model included six prognostic factors: age, primary site, summary stage, surgery, tumor size, and chemotherapy. The nomogram was then evaluated using C-index, and the C-index for predicting CSS in the training cohort and validation cohort in this study was 0.795 and 0.812, respectively. When the C-index range was between 0.5 and 1.0, the predicted survival was found closer to the actual survival (15). Moreover, the AUC values of the ROC curves were found to be considerably elevated (>0.7), as shown in Figure 4, further supporting that the nomogram has high prediction accuracy. The calibration plots also showed a good agreement between the prediction results and the actual observed results. The findings of this investigation support the substantial prediction accuracy of our generated nomogram, which is both internally and externally validated. It allows doctors and patients to determine the likelihood of cancer-specific survival at 1, 3, and 5 years, which can help them decide on the best course of therapy and follow-up schedule.

The current study associated with some limitations: Firstly, the SEER dataset does not have effective biological factors, including MYCN status, DNA index (ploidy), the allelic status of chromosome 11q23, and some prognosis-related variables, such as surgical margin status, staging data for INSS and INRGSS, and detailed radiotherapy and chemotherapy plans. Second, due to the study's retrospective nature, it is inevitable that some patient data would be lost. This will lower the number of valid cases and could result in selection bias. Third, external validation of our prediction model with another set of independent, large-scale data would have made the conclusions of this study more reliable and broadly applicable. Despite these limitations, the nomogram we constructed has the largest sample size, the included indicators are all clinically accessible, and it is currently the most accurate and reliable model capable of predicting survival outcomes in children with NB. In the future, a better experimental design without the above limitations will be needed to facilitate clinical application.

## Conclusion

Taken together, a novel nomogram was established for predicting the 1-, 3-, and 5-years CSS rates of NB patients, based on the SEER database of 1,448 children with neuroblastoma. This analysis demonstrates that our developed nomogram has a high degree of predictive accuracy. It enables physicians and patients to assess the probability of cancer-specific survival at 1, 3, and 5 years, which can guide them in determining the optimal course of treatment and follow-up plan.

## Data Availability

The original contributions presented in the study are included in the article/supplementary material, further inquiries can be directed to the corresponding author/s.
